# Assessing the efficacy of machine learning algorithms for syncope classification: A systematic review

**DOI:** 10.1016/j.mex.2023.102508

**Published:** 2023-12-06

**Authors:** Choon-Hian Goh, Mahbuba Ferdowsi, Ming Hong Gan, Ban-Hoe Kwan, Wei Yin Lim, Yee Kai Tee, Roshaslina Rosli, Maw Pin Tan

**Affiliations:** a Department of Mechatronics and BioMedical Engineering, Lee Kong Chian Faculty of Engineering and Science, Universiti Tunku Abdul Rahman, 43000 Kajang, Selangor, Malaysia; b Centre for Healthcare Science and Technology, Universiti Tunku Abdul Rahman, 43000 Kajang, Selangor, Malaysia; c Electrical and Computer Systems Engineering, School of Engineering and Advanced Engineering Platform, Monash University Malaysia, Bandar Sunway 47500, Selangor, Malaysia; d ACT4Health Services and Consultancy, 47300 Petaling Jaya, Malaysia; e Ageing and Age-Associated Disorders Research Group, Department of Medicine, Faculty of Medicine, Universiti Malaya, 50603 Kuala Lumpur, Malaysia; f Department Medical Sciences, Faculty of Healthcare and Medical Sciences, Sunway University, 47500 Bandar Sunway, Malaysia

**Keywords:** Syncope diagnosis, Machine learning, Classification, Systematic review, Methodology for conducting a systematic literature review

## Abstract

Syncope is a transient loss of consciousness with rapid onset. The aims of the study were to systematically evaluate available machine learning (ML) algorithm for supporting syncope diagnosis to determine their performance compared to existing point scoring protocols. We systematically searched IEEE Xplore, Web of Science, and Elsevier for English articles (Jan 2011 - Sep 2021) on individuals aged five and above, employing ML algorithms in syncope detection with Head-up titl table test (HUTT)-monitored hemodynamic parameters and reported metrics. Extracted data encompassed subject count, age range, syncope protocols, ML type, hemodynamic parameters, and performance metrics. Of the 6301 studies initially identified, 10 studies, involving 1205 participants aged 5 to 82 years, met the inclusion criteria, and formed the basis for it. Selected studies must use ML algorithms in syncope detection with hemodynamic parameters recorded throughout HUTT. The overall ML algorithm performance achieved a sensitivity of 88.8% (95% CI: 79.4–96.1%), specificity of 81.5% (95% CI: 69.8–92.8%) and accuracy of 85.8% (95% CI: 78.6–92.8%). Machine learning improves syncope diagnosis compared to traditional scoring, requiring fewer parameters. Future enhancements with larger databases are anticipated. Integrating ML can curb needless admissions, refine diagnostics, and enhance the quality of life for syncope patients.

## Review Highlights

• The first systematic review on syncope classification using machine learning (ML) algorithms includes an evaluation of ML algorithm performance in predicting syncope based on hemodynamic parameters.
• The machine learning algorithms have demonstrated their capability to detect syncope with a sensitivity of 88.8%, specificity of 81.5%, and an overall accuracy of 85.8%.
• Integration of ML in syncope diagnosis holds the potential to simplify diagnosis with a reduced set of parameters for more efficient assessments and, as a result, reduce unnecessary hospital investigations.

## Graphical abstract

**Figure:**
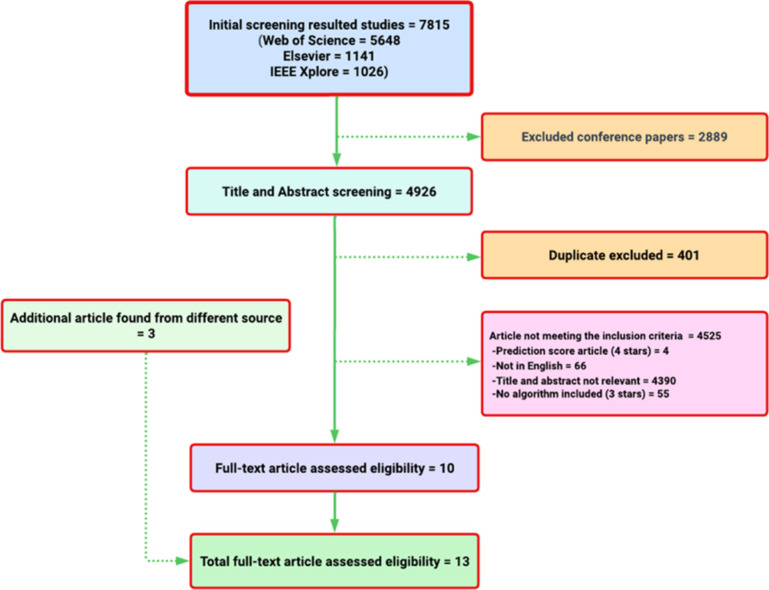

Specifications table

**Table:** 

Subject area:	Engineering
More specific subject area:	Biomedical Engineering
Name of the reviewed methodology:	Methodology for conducting a systematic literature review
Keywords:	Syncope diagnosis; machine learning; classification; systematic review
Resource availability:	Not applicable
Review question:	1. What are the different types of machine learning (ML) algorithms that have been used for syncope diagnosis, particularly those incorporating physiological signals and what are the key features extracted from these signals for diagnosis? 2. What are the different types of ML algorithms that have been used for syncope diagnosis? 3. What is the overall performance of these ML algorithms in terms of accuracy, sensitivity, specificity, and other relevant metrics, and how does this performance compare to existing point scoring protocols?

## Method overview

### Introduction

Syncope is defined as transient loss of consciousness (TLOC) due to global cerebral hypoperfusion, which is characteristically of rapid onset, brief duration with complete spontaneous recovery [1,2]. It is a common condition, with 18.9–39.7 per 1000 patient episodes reported in the general population [1]. The Framingham Heart Study reported an overall incidence rate of 6.2 per 1000 person-years with increased incidence with age, and a sharp increase after 70 years [3]. An incidence rate of 11.1 per 1000 person-years has been assigned to those aged 70–79 years and 18.25 per 1000 person-years for those aged 80 years and above [4]. Approximately 40% of the U.S. population experienced a syncopal episode in their lifetimes, with 30–50% admitted to the hospital for further evaluation, and one-third of cases were classified with an unexplained etiology [5].

Syncope can be classified into three main types: neurally-mediated or neurocardiogenic or reflex, orthostatic hypotension and cardiac syncope. While syncope has a relatively low mortality rate overall, the mortality rate rises sharply with increasing age. An annual mortality rate of 14% has been reported in individuals patients aged 70–79 years, increasing to 22% in patients aged 80–89 years, and 43% in patients aged ≥90 years [6]. Syncope, however, is associated with increased healthcare utilization, accounting for up to 6% of all hospitalizations and 3% of all emergency room visits in hospitals [7]. Syncope is also known to affect the quality of life, interfering with the activities of daily life with potential occupational implications [8]. Besides physical injuries and disabilities due to syncope-related falls, older individuals with syncope may also develop depression and reduced functional capacity with consequent institutionalization [6].

Diagnostic strategies for syncope may include head-up tilt table test (HUTT) and implantable loop recorder (ILR) [9], [10], [11], [12]. However, the gold standard diagnostic techniques for syncope, exemplified by the HUTT present inherent limitations such as extended test duration, invasiveness, and potential discomfort [13]. Additionally, these methods may lack sensitivity, leading to inaccuracies in diagnoses and false negatives [14]. Despite efforts to discontinue the HUTT promptly upon symptom onset, it remains unsuitable for physically weak patients and can evoke unpleasant experiences. Moreover, [15] reported sensitivities of 32% and 85%, with the median closer to the upper number, suggest potential high false negative rates and subsequent misdiagnoses. Recognizing these challenges and the impact of syncope on healthcare, hospitalizations, and well-being, this study conducts a systematic review.

The aim is to compare syncope assessment using the HUTT with early detection algorithms rooted in machine learning (ML). The objective is to assess the suitability and precision of ML algorithms for enhancing diagnostic capabilities in clinical and hospital settings, particularly focusing on vulnerable populations.

Early syncope detection using machine learning (ML) algorithms is gaining popularity with the promise of avoidance of provoking unpleasant symptoms. Early syncope detection may potentially reduce morbidity with early accurate diagnosis which inevitable leads to the delivery of prompt treatment. By using ML algorithms to predict the outcome of HUTT, it is postulated that the overall procedure will be faster compared to traditional HUTT tests, potentially increasing the use of HUTT, enhancing diagnostic capacities.

In this study, a systematic review is conducted to compare the assessment of syncope using HUTT procedure with early detection algorithms using to determine the suitability and accuracy of ML algorithms for implementation in the clinical and hospital field. Methods of early detection are also discussed, and comparisons made between prediction using ML algorithms and other protocols including risk scores.

## Methods details

### Data sources and searches

Articles related to syncope detection by using ML algorithms were identified from the databases IEEE Xplore, Web of Science and Elsevier using the search terms (syncope OR passing out) AND (detect* OR diagnosis). Only English language articles were chosen.

### Study selection

The PICO (Patient, Intervention, Comparison, Outcomes) approach was followed. Articles were selected based on the following inclusion criteria: (i) Research article: published from Jan 1, 2011, to September 30, 2021; (ii) Population: children (aged 5 years old) to adults (no upper age limit); (iii) Outcomes: studies which employed ML algorithms in syncope detection using hemodynamic parameters recorded throughout HUTT, which reported performance metrics.

Articles which met the following criteria were excluded: (i) syncope detection algorithm based on scoring metrics; (ii) syncope detection algorithm based on hemodynamic parameters collected from ILR.

### Data extraction

The data extraction process involved two reviewers (M.F & M.H. Gan) who extracted the data from selected articles independently, with any discrepancies being resolved through discussion. A third reviewer (C.H. Goh) was consulted if disagreements occurred.

### Quality assessment

The ChAMAI (Checklist for Assessment of Medical Artificial Intelligence) formally known as IJMED checklist, a tool for assessing the quality of medical artificial intelligence studies [13] was used in this study. The tool was used independently by two reviewers, MF and M.H. Gan but if there were any discrepancies, the final decision was made after discussion with C.H. Goh. The checklist comprises six parameters, named as problem understanding, data understanding, data preparation, modelling, validation, and deployment, and consist of a total of 30 questions. The article-review support tool includes four options for evaluation, namely NA (not applicable), OK (adequately addresses), mR (minor revisions needed), and MR (major revisions needed). For high-priority items, each question is assigned a score of 2, 1, 0 for OK, mR, and MR, respectively, whereas for low-priority items, the scores are halved 1, 0.5, and 0. The maximum score shall be 50 points, but a lower total score was allocated if there are certain NA items. The quality of the studies was classified as low (0–39.9%), medium (40–69.9%) or high (70–100%) based on the percentage of total score.

### Outcomes

The outcomes considered were the performance metrics including sensitivity, specificity, and accuracy of ML classification algorithms on the early detection of syncope. Techniques of ML algorithms used were identified, the application of drugs on the HUTT were recorded, and the performance metrics of ML algorithms and scoring methods were compared.

### Statistical analysis

Forest plots serve as graphical representations of the outcomes in systematic reviews [14]. In this context, a forest plot was generated, incorporating findings from thirteen selected studies for comprehensive analysis. For each individual study, sensitivity, specificity, and accuracy with its 95% confidence interval (CI) was calculated for every predefined outcome. The highest performance of the algorithm of each study was included into the forest plot analysis and the averaged performance and confidence interval was computed. The formulae used to derive all the values was obtained from [15], and as shown as below:

(1) $$95\%\;CI\;of\;Sensitivity=\;Sensitivity\;\pm \;1.96\;\left(\sqrt{\frac{Sensitivity-\left(1-Sensitivity\right)}{n_{Sensitivity}}}\right)$$

(2) $$95\%\;CI\;of\;Specificity=\;Specificity\;\pm \;1.96\;\left(\sqrt{\frac{Specificity-\left(1-Specificity\right)}{n_{Specificity}}}\right)$$

(3) $$95\%\;CI\;of\;Accuracy=\;Accuracy\;\pm \;1.96\;\left(\sqrt{\frac{Accuracy-\left(1-Accuracy\right)}{n_{Accuracy}}}\right)$$

where,

• $n_{sensitivity}\;=\;total\;positive\;patient$
• $n_{specificity}\;=\;total\;negative\;patient$
• $n_{total\;}=\;total\;patient$
  (4) $$Sensitivity\;=\;\frac{TP}{TP+FN}$$
  (5) $$Specificity\;=\;\frac{TN}{TN+FP}$$
  (6) $$Accuracy\;=\;\frac{TP+TN}{TP+TN+FP+FN}$$

Where, TP = True Positive; FN = False Negative; TN = True Negative; FP = False Positive

## Analysis

### Study selection

**Fig. 1:**
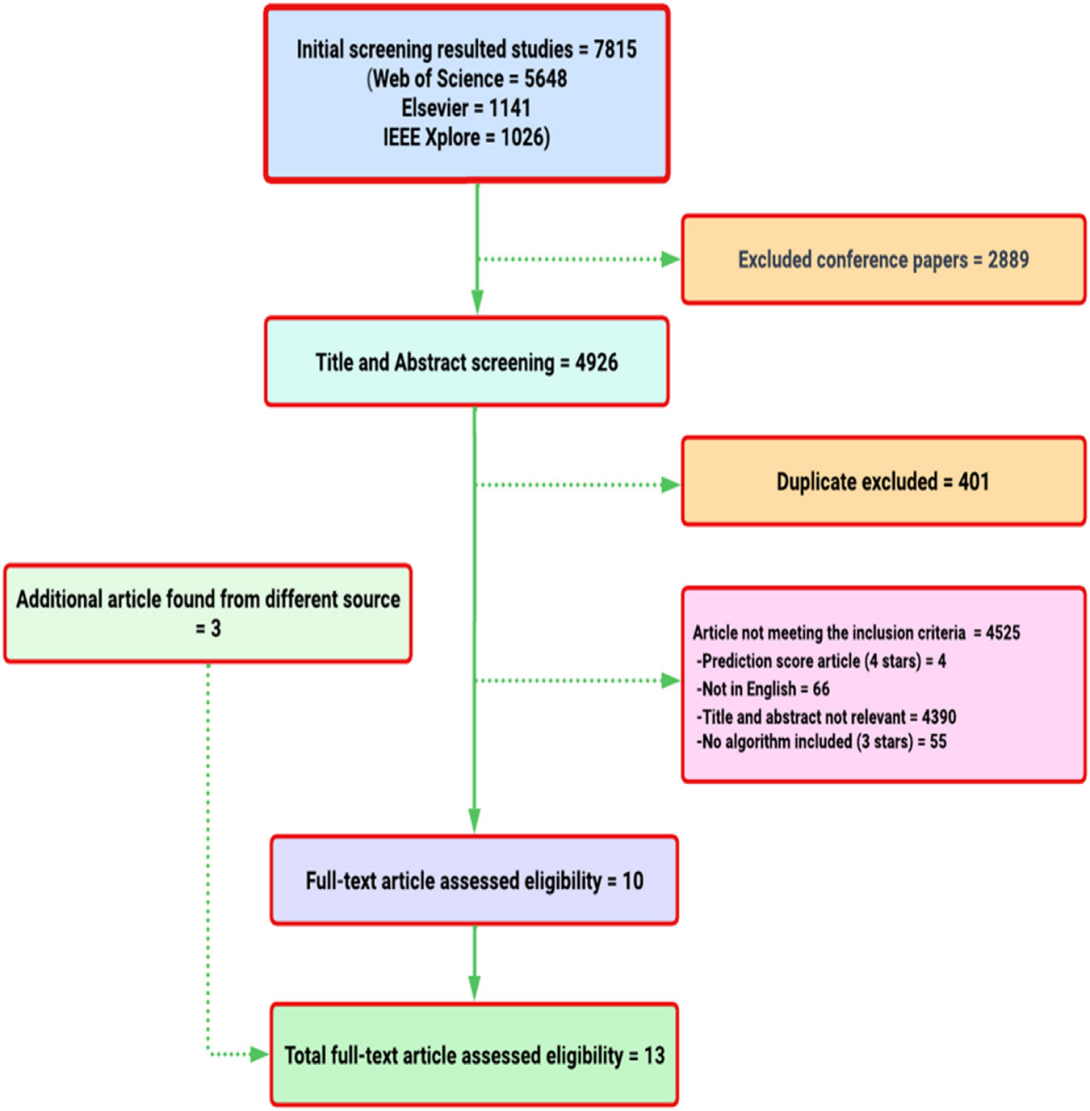
Flowchart of the Study Selection. A flowchart that employing the PRISMA diagram to visualize the systematic search process. A total of 7815 articles were identified from three major databases (5648 from Web of Science, 1141 from Elsevier, and 1026 from IEEE Explorer). Subsequently, ten articles were selected, with an additional three from different sources.

From three major databases, a total of 7815 articles (5648 from Web of Science; 1141 from Elsevier; 1026 from IEEE explorer) were identified. Of these, 2889 were excluded as they were not journal articles (e.g., conference paper), leaving 4926 articles for title and abstract screening. 401 of these articles were duplicated and were therefore eliminated. A further 4525 were excluded after their titles and abstracts were reviewed. Of these 4390 were not relevant to the topic and 66 were English language articles. The full text versions of the remaining articles were then reviewed, following which 55 articles were further excluded, leaving 10 selected articles. Another 3 articles from different source were included. Fig. 1 represents the PRISMA diagram for the systematic search process.

### Assessment of study quality

**Fig. 2:**
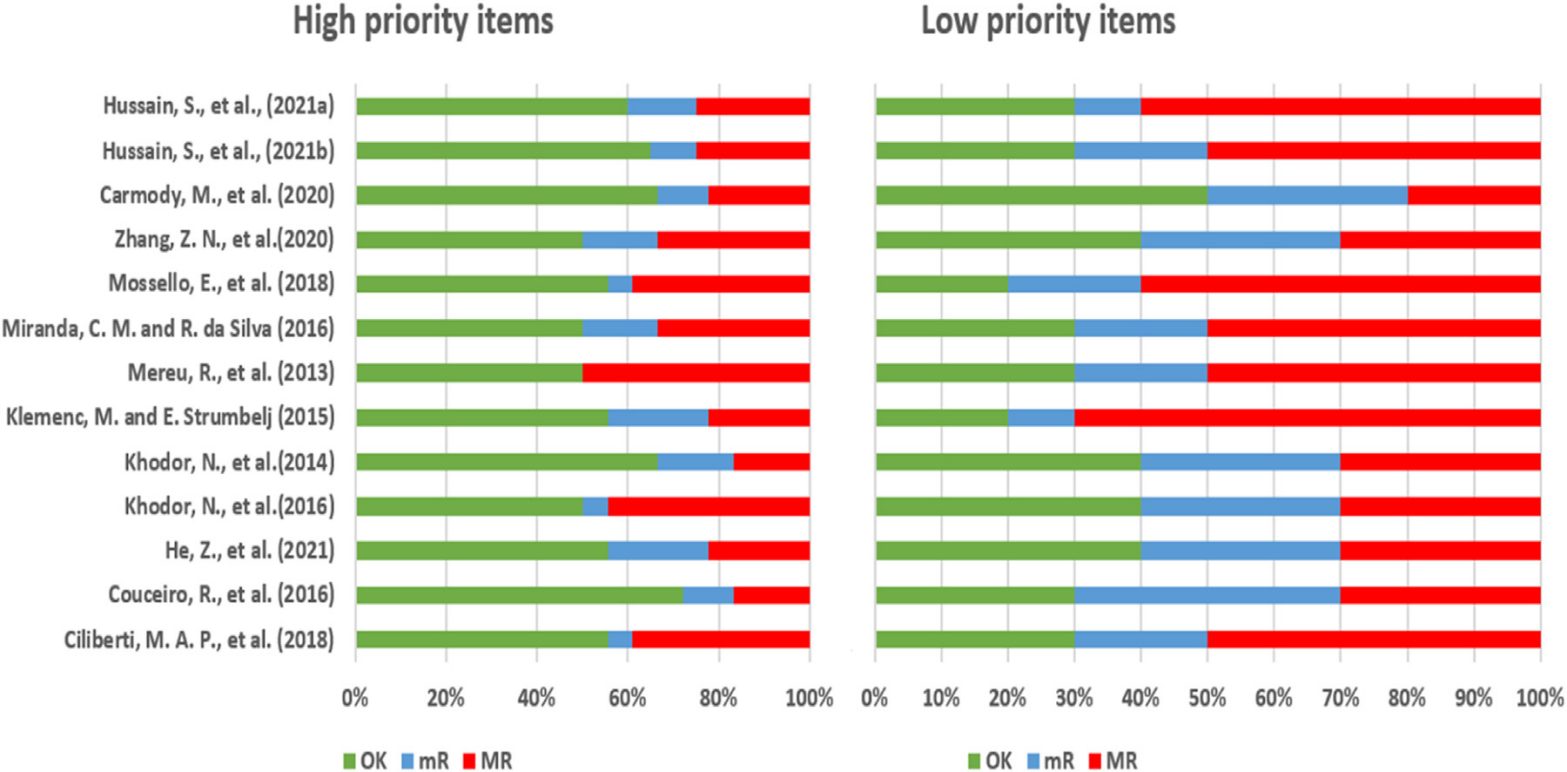
Proportion of the different answers in the high- and low-priority items. The comparison of checklist scores between low-priority and high-priority parameters, employing a 50-point scale and a four-tier evaluation system (NA - not applicable, OK - adequately addresses, mR - minor revisions needed, and MR - major revisions needed). The figure showcases the distribution of scores across six checklist parameters, emphasizing the impact of double weighting on high-priority items and classifying the overall study quality as low, medium, or high.

The quality assessment performed according to the criteria developed by ChAMAI checklist. Table 1 summarizes the scores of each dimension and the total score in each study. Three (Couceiro et al., 2016, Khodor et al., 2014, Carmody et al., 2020) out of thirteen papers were considered as high-quality paper, while the rest papers were of a medium quality. Fig. 2 shows the proportion of the different answer in the high- and low-priority items.

**Table 1:** Quality assessment scores of 13 studies according to the ChAMAI checklist.

Authors (Year)	Problem understanding [10]	Data understanding [6]	Data preparation [6]	Modeling [6]	Validation [12]	Deployment [8]	Total of 100% (%)
He, Z. et al (2021) [18]	8	4	2	6	7	2.5	64.13
Hussain, S. et al. (2021a) [19]	9	3	4*(8)	6	7	1.5	61
Hussain, S. et al. (2021b) [26]	9	2	6 *(8)	6	7	2	64
Carmody, M., et al. (2020) [16]	10	5	0	6	9	2.5	70.65
Zhang, Z. N. et al. (2020) [23]	10	2	0	6	5.5	3	57.61
Mossello, E. et al. (2018) [40]	9	5	0	6	3	1	52.17
Ciliberti, M. A. P., et al. (2018) [31]	10	5	0	6	3	1	54.35
Miranda, C. M. and R. da Silva (2016) [36]	10	4	1	6	3	1	54.35
Khodor, N. et al. (2016) [20]	10	0	2	6	3	2.5	51.09
Couceiro, R., et al. (2016) [17]	9.5	4	4	6	8	2.5	73.91
Klemenc, M. et al. (2015) [22]	10	5	3	6	2	0.5	57.61
Khodor, N. et al. (2014) [21]	10	4	2	6	8	2.5	70.65
Mereu, R., et al. (2013) [30]	9	2	0	6	3	2	47.83

### Analytical strategies

Three types of signals were recorded during HUTT including ECG, Blood Pressure (BP), and Photoplethysmography (PPG). Some of the prediction models have conducted feature selection by using different techniques such as Mann-Whitney U test [16], scoring [17], Genetic Algorithm (GA) [18], Principle Component Analysis (PCA) [19], relief method [20], Sequential Forward Selection (SFS) [20], probe feature algorithm(20), Detrended Fluctuation Analysis (DFA) [21], Sample Entropy (SampEn) [21], linear regression [22] and multivariate logistic regression [23].

Several common parameters were extracted from the collected signals including Heart Rate Variability (HRV), RR-Interval (RRI), Diastolic Blood Pressure (DBP), Systolic Blood Pressure (SBP), Mean Blood Pressure (MBP). Heart Rate (HR), Cardiac Output (CO), Stroke Volume (SV), Total Peripheral Resistance (TPR) and Left Ventricular Ejection Time (LVET). Seven studies have carried out cross-validation, which were [17] and [22] using leave-one-out cross-validation, (Hussain et al., 2021a) and (Hussain et al., 2021b) using ten-fold cross-validation, [18] using five-fold cross-validation while [21] and [20] using two-fold cross-validation.

### Machine learning classifiers

The researchers in this syncope classification study used several ML classification algorithms, namely Support Vector Machine (SVM), K-Nearest Neighbors (KNN), Multinomial Naive Bayes (MNB), Gaussian Naive Bayes (GNB), Logistic Regression (LR), Random Forest (RF), and Decision Tree (DT).

I. Support Vector Machine

Support Vector Machine (SVM) is the construction of a maximum marginal hyperplane that divides the dataset into classes as evenly as possible. The input data was non-linearly transferred to a high-dimensional space using kernels. The study accustomed the linear or radial basis function (RBF) kernel [24]. Any two measures can be combined using a linear kernel's normal dot product. It is the result of multiplying all the input values together.

(7) $$k\left(x,x_{i}\right)=sum\left(x*x_{i}\right)$$

RBF is dimensionally unbounded. Any input can be mapped by RBF into any dimensional space.

(8) $$k\left(x,x_{i}\right)=exp\left((-gamma*sum\;(x*x_{i}^{2}\right)\;$$

II. K Nearest Neighbors

K Nearest Neighbors (KNN) is a non-parametric method. The distance metric employed in this method, called Minkowski distance, can only be calculated in a normed vector space, which is a space in which dimensions can be expressed by vectors whose lengths cannot be negative. Minkowski distance is represented by the equation below:

(9) $$L\left(xi,xj\right)={\left(\sum_{i,j=1}^{n}{\left(\;\left(\left|xi-xj\right|\right)\right)}^{2}\right)}^{1/2}\;X\;\in \;\text{Rn}\;$$

The KNN algorithm's parameter k determines how many neighbours will be chosen. The diagnostic effectiveness of the KNN algorithm is greatly influenced by the choice of k [25]. In a KNN, the number of samples in the training set may have the highest k value. However, employing such a high number for “k” would lead to a highly smooth and overly broad decision boundary that might not be a good fit for the underlying data. The number of nearest neighbours to consider for voting is indicated by the term “n_neighbors”. It defines how many of the closest neighbours were considered when classifying a fresh sample by the majority. The power parameter for the Minkowski distance metric is denoted by “p”.

III. Multinomial Naïve Bayes

Multinomial Naïve Bayes (MNB) is referred as a probabilistic learning approach based on the Bayes Theorem, where the features are assumed to be independent of each other. When it comes to HUTT data, MNB can be utilized to assess the probability of a patient experiencing syncope, s, amongst the class of patients, c, as [26]

(10) $$P\left(c\;s\right)\propto \;\prod_{1\leq k\leq \text{ns}\;}P(x_{k}\left|\;c\right)$$

Here P $\left(x_{k}\right|$c) represents the conditional probability of feature $x_{k}$ occurring in a dataset of the class of patient c. P $\left(x_{k}\right|$c) measures the contribution of feature $x_{k}$ in finding the correct class c. P(c) is the prior probability of the occurrence of syncope in class c. When features don't clearly distinguish between one class and another, the class with the larger prior probability is picked. The number of variables considered for classifications is represented by ns. Both continuous and discrete datasets can be used with this approach.

IV. Gaussian Naïve Bayes

In handling continuous data, a typical assumption is that the continuous values for each class will exhibit a Gaussian distribution in Gaussian Naïve Bayes (GNB).For instance, that the class label c and the continuous nature of the $i^{th}$ characteristic result in mean and variance are being represented by $\mu_{c,i}$ and $\sigma_{c,i}^{2}$ respectively. The likelihood of seeing the value $x_{i}$ in the ith attribute given the class label c is, therefore, calculated using an equation commonly known as the normal distribution [26].

(11) $$p\left(xi|c\right)=\frac{1}{\sqrt{2\pi \sigma_{c,i}^{2}}}\;\exp\left(-\frac{{\left(xi-\mu_{c,i}\right)}^{2}}{2\sigma_{c,i}^{2}}\right)$$

V. Logistic Regression

Logistic Regression (LR) is employed to predict the frequency of a target attribute. Only two valid classes exist because the dependent or target variable is dichotomous. The dependent variable is, by definition, a binary variable, meaning that data can only take the values 1 or 0. For the categorization of syncope, the class variable Y = 1, 2, and the d-dimensional feature vector X = ($X_{i}$,..., $X_{d}$) are used. Let p1(x) stand for the likelihood that Y = 1 given that X = x. Assumptions are made in the binary logistic regression model as [27]

(12) $$p1\left(x\right)=\frac{1}{1+\text{exp}\left[-\left(\beta^{T}x+\beta_{0}\right)\right]}\;$$

Generally, parameters β and $\beta_{0}$are estimated by maximizing the conditional log-likelihood given a learning set ($x_{i}$, $y_{i}$) with $n_{i=1}$

(13) $$l\left(\beta ,\beta_{0}\right)=\sum_{i=1}^{n}y_{i}1\ln p1\;\left(x_{i}\right)+\left(1-y_{i}1\right)\text{ln}\left[1-p1\left(x_{i}\right)\right]$$

where $y_{i}$ = 1 if $y_{i}$ is less than 1 and 0 otherwise.

VI. Random Forest

The Random Forest (RF) classifier is made up of several tree classifiers. With each tree being created using the training set and the random vector k, the random forest algorithm creates a collection of classifiers using a tree structure called h (x, k), where k = 1 and is distinct from the at vector input x. Generalization error in the random forest algorithm is provided as [28]

(14) $$PE=P_{X,Y\left(mg\left(X,Y\right)<0\right)}$$

where mg is marginal function, which determines how much the total number of votes cast using randomized vectors for the desired outcome is greater than the average vote for all other desired outcomes, and subscripts X and Y are random vectors that indicate the probability is across the X, Y space. The margin function can be defined as

(15) $$mg\left(X,Y\right)=av_{k}I\left(h_{k}\left(X\right)=Y\right)-max_{j\neq Y\;}av_{k}\;I\left(h_{k}\left(X\right)=j\right)$$

the indicator function I is included. Because features are randomly selected, reducing correlation between trees in the ensemble, this approach often enhances the predictive capacity of the ensemble.

VII. Decision Tree

A Decision Tree (DT) is comprised of decision tests employing a divide-and-conquer approach, forming a tree structure. It includes leaf nodes and branches, with the root node positioned at the tree's top. Nodes represent feature tests used for data division, while leaf nodes indicate data labels, and branches depict the routes based on test outcomes. This recursive method divides the data into subsets in each step, using each subset for subsequent phases, determined by the chosen split [29].

### Performance metrics

Ten studies reported the sensitivity and specificity of their algorithm for early detection of syncope while one study [22] did not mention the sensitivity and specificity of algorithms, two study (Hussain et al., 2021a) and (Hussain et al., 2021b) did not mention the specificity of algorithms. However, only two studies specified the accuracy of their algorithms, and five studies reported the Positive Predictive Value (PPV). Fig. 2 represents the forest plot of sensitivity, specificity and PPV extracted from the selected studies where possible. From Table 2, the range of the sensitivities was 43.4–97.8%, the range of specificities was 56–97.3%, the range of accuracy was 57.5–98.9%, and the range of PPV was 75–91.7%.

**Table 2:** Characteristics of each included study.

Article	Subjects		Age range	HUTT protocol	Type of signals	Feature Selection algorithm	Feature Selected	Classification algorithm	Performance Metrics			
	No. Subjects (n)	Men (n, %)							Sensitivity (%)	Specificity (%)	Accuracy (%)	PPV (%)
Carmody, M., et al. (2020)	101	-	14-39	Initially active stand then HU TT (5 mins supine + 20 mins 70° + GTN)	PPG	Mann Whitney U test	SBP-5s%, SBP-20s%, HR-%I, CO- %I, SV-B2SS	Univariate analysis, multivariable analysis	84.3	72.9	80.2	84.4
Ciliberti, M. A. P., et al. (2018)	26	11 (42.3)	21 - 58	HUTT (30 mins resting state + 45 mins 60 degree) + (15mins NTG)	ECG, BP	-	HRV, VLF, LF, HF, LF/HF ratio	Univariate analysis, multivariable analysis, logistic regression	87.5	72.2	76.9*	75
Couceiro, R., et al. (2016)	43	23 (53.5)	39 - 80	HUTT (15 mins lying rest + 20mins 70 degree) + (15mins NTG)	ECG, PPG	Feature selection score	PAT, SI, HR, LVET, RI	ROC analysis	95.2	95.4	95.4*	90.9
He, Z., et al. (2021)	209	76 (36.4)	22.4 - 61.4	HUTT (5 mins supine + 20mins 70 degree) + (15 mins NTG)	ECG	GA	HR, RRI, SBP, DBP, MBP, LVET, TPR, CO, SV	SVR, LR, KNN, RF	SVR: 86 LR: 82 KNN: 84 RF: 81	SVR: 82 LR: 71 KNN: 81 RF: 79	SVR:84.2* LR: 63.2* KNN: 83.3* RF:80.3*	-
Hussain, S., et al., (2021a)	687	-	-	HUTT (20 mins supine + 20 mins 60° to 80°)	BP, ECG	PCA	SBP, DBP, TPR, HR	SVM, KNN, SGD	SVM: 92.2 KNN: 43.4 SGD: 77.8	-	SVM: 97.5 KNN: 90.8 SGD: 83.2	-
Hussain, S., et al., (2021b)	687	-	-	HUTT (20 mins supine + 20 mins 60° to 80°)	BP, ECG	-	BeatStats, Cardiac BeatStats, HRV Stats, dBPV Stats, sBPV Stats	DT, MNB, GNB, KNN, SVM, LR	LR: 97.6 DT: 86.7 GNB: 93.9 KNN: 43.2 MNB: 97.8 SVM: 90.9	-	LR: 98.9 DT: 97.8 GNB: 95.9 KNN: 91.4 MNB: 57.5 SVM: 97.5	-
Khodor, N., et al.(2016)	57	-	18 - 35	HUTT (15 mins supine + 45 mins 80 degree)	ECG, BP	Relief method, SFS, Probe feature algorithm	RRI, Amps, dPdt_max, PTT	KNN, SVM	KNN: 86.4 SVM: 87.5	KNN: 87.9 SVM: 93.8	KNN: 86.0* SVM: 89.5*	87.5
Khodor, N., et al.(2014)	66	-	18 - 35	HUTT (11 mins supine + 45 mins 80 degree)	ECG, BP	DFA, SampEn	RRI, SS-interval	KSVM	88.5	80.6	84.8*	-
Klemenc, M. et al. (2015)	92	38 (41.3)	16 - 82	HUTT (5 mins stabilization + 45 mins 65 degree + 5 mins final) + (15 mins NTG)	ECG, BP	Linear regression	HRV, BRS, RRI	Logistic regression	-	-	80.6*	-
Mereu, R., et al. (2013)	145	59 (40.7)	7 - 82	HUTT (5 mins supine + 35mins 60 degree)	ECG, BP	-	RRI, SBP, DBP, MBP, RR/SBP, dRR/SBP, dRR/DBP, dRR/MBP, dRR/PP	ROC analysis with classification	RRI: 84.4 SBP:88.9 DBP:87.4 MBP:86.2 RR/SBP:52.8 dRR/SBP:86.2 dRR/DBP:61.2 dRR/MBP:80.6 dRR/PP:82.0	RRI: 74 SBP:67.2 DBP:79.5 MBP:72.7 RR/SBP:97.3 dRR/SBP:89.1 dRR/DBP:93.2 dRR/MBP:86.4 dRR/PP:93.2	RRI:78.6* SBP: 77.9* DBP: 83.4* MBP: 79.3* RR/SBP: 74.5* dRR/SBP: 87.6* dRR/DBP: 67.6* dRR/MBP: 83.4* dRR/PP: 87.6*	-
Miranda, C. M. and R. da Silva (2016)	64	35 (54.7)	14 - 77	HUTT (10 mins supine+20 mins 70 degree) + (15 mins isosorbide)	ECG	-	HRV, LF, HF, LF/HF	ROC analysis	97.4	83.3	92.2*	85.3
Mossello, E., et al. (2018)	372	146 (39.2)	>65	HUTT (5 min supine + 20 min 60 degree) +(NTG)	ECG, BP	-	-	Multinomial logistics regression	82	56	75.9*	-
Zhang, Z. N., et al.(2020)	176	86 (48.9)	5 - 17	HUTT (Duration not specified)	ECG, BP	Multivariate logistic regression with forward selection	SBP, DBP, HR	Logistic regression	89.3	80.8	90.9*	91.7

⁎ Accuracy is back-calculated, PPV: positive predictive value, HUTT: head-up tilt test, ECG: electrocardiogram, BP: blood pressure, HRV: heart rate variability, VLF: very low frequency, LF: low frequency, HF: high frequency, PPG: photoplethysmography, LVET: left ventricular ejection time, SI: stiffness index, PAT: pulse arrival time, RI: reflection index, ROC: receiver operating characteristic, GA: genetic algorithm, RRI: R-R interval, SBP: systolic blood pressure, DBP: diastolic blood pressure, MBP: mean blood pressure, TPR: total peripheral resistance CO: cardiac output, SV: stroke volume, SVR: support vector regression, LR: logistic regression, KNN: k-nearest neighbour, RF: random forest, SFS: sequential forward selection, Amps: point on the BP, dPdt_max: point on the dP/dt signal, PTT: pulse transit time, KSVM: kernel support vector machine, DFA: detrended fluctuation analysis, SampEn: sample entropy, BRS: baroreflex sensitivity, PP: pulse blood pressure.

The highest sensitivity was 97.8% (Hussain et al., 2021b) which the highest specificity reported was 97.3% [30]. achieved the highest accuracy at 98.9% and the highest PPV achieved by Zhang et al. [23] at 91.7%. Although Hussain et al., 2021b achieved highest sensitivity, their specificity did not report and unable to calculate backward as their confusion matrix provided unable to compute specificity. The RR/SBP variable from Mereu et al. [30] achieved the highest specificity but the lowest sensitivity with 52.8%. However, the algorithm from Mereu et al. was not considered the best performing studies due to the trade-offs. Since Hussain et al. achieved the highest sensitivity and accuracy, it considered as the best performing study.

**Fig. 3:**
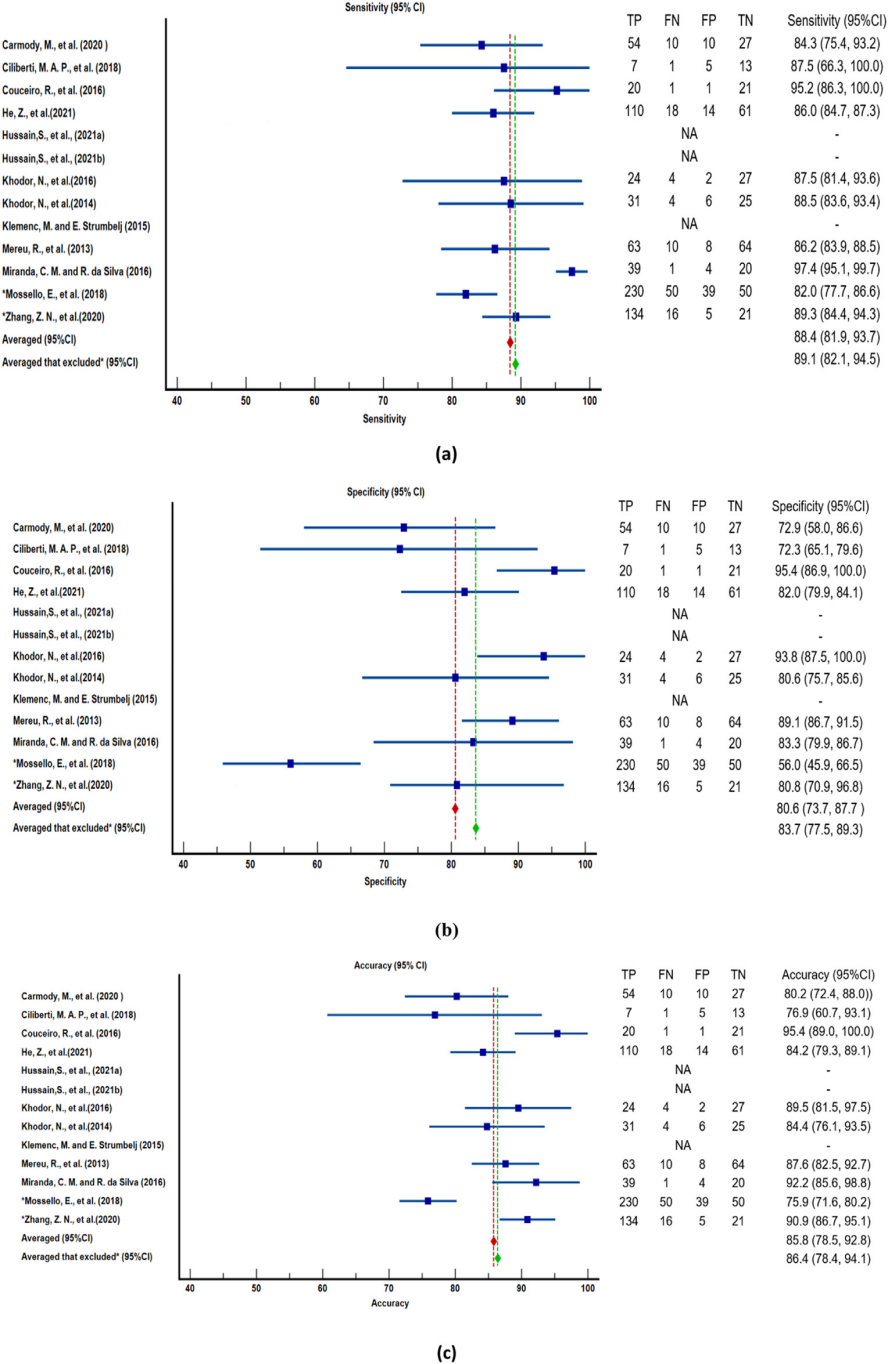
Forest Plot of Performance Metrics. It is showcasing (a) Sensitivity, (b) Specificity, and (c) Accuracy for machine learning algorithms in Syncope classification across selected studies. The comprehensive forest plot provides a visual overview of performance metrics, including estimates and corresponding 95% confidence intervals. This graphical representation enables a quick assessment of variability and precision across multiple studies.

By referring Fig. 3, there has two average performance matric such as sensitivity, specificity and accuracy reported as one of the average values are included all the 13 studies while the second average has excluded the studies that labelled *, which are Mossello et al. and Zhang et al. The purpose of excluding these two studies is the target of study is not same category with the other studies as for Zhang et al is pediatric patient and for Mossello et al is patient that above 65 years of age, where the rest of the studies has a larger age range. Combining all the studies and calculate the mean value of the sensitivity, specificity and accuracy might causing bias. Thus, two different ways to calculate the averaged and the value were reported.

### Effect of provocation agents on head-up tilt table test

From the Table 2 assessment method column, seven out of thirteen studies mentioned that provocation agents were used during the HUTT assessment. The drugs that were used in HUTT included nitroglycerin (Glyceryl Trinitrate, GTN) and isosorbide. The use of provocation agents did not appear to influence the sensitivity and specificity of algorithms. For example [31], [Sensitivity: 87.5 %, Specificity: 72.2 %] and [21] [Sensitivity: 88.5 %, Specificity: 80.6 %] had similar performance metrics although [21] did not use provocation agents for HUTT assessment. However, Couceiro et al. [17] [Sensitivity: 95.2 %, Specificity: 95.4 %] obtained the best sensitivity-specificity compromise with drug application.

The sensitivity range for HUTT with potentiating agents varied from 81 % to 97.4 % with the mean value of 86.6 % while specificity varied from 56 % to 95.4 % with the mean value of 77.0 %. Without drug application, the sensitivity of mean value was 81.13 % with a range of 43.2–97.8 % while mean value for specificity was 84.28 % with a range of 67.2–97.3 %.

### Comparison of machine learning algorithms and clinical risk scores

**Fig. 4:**
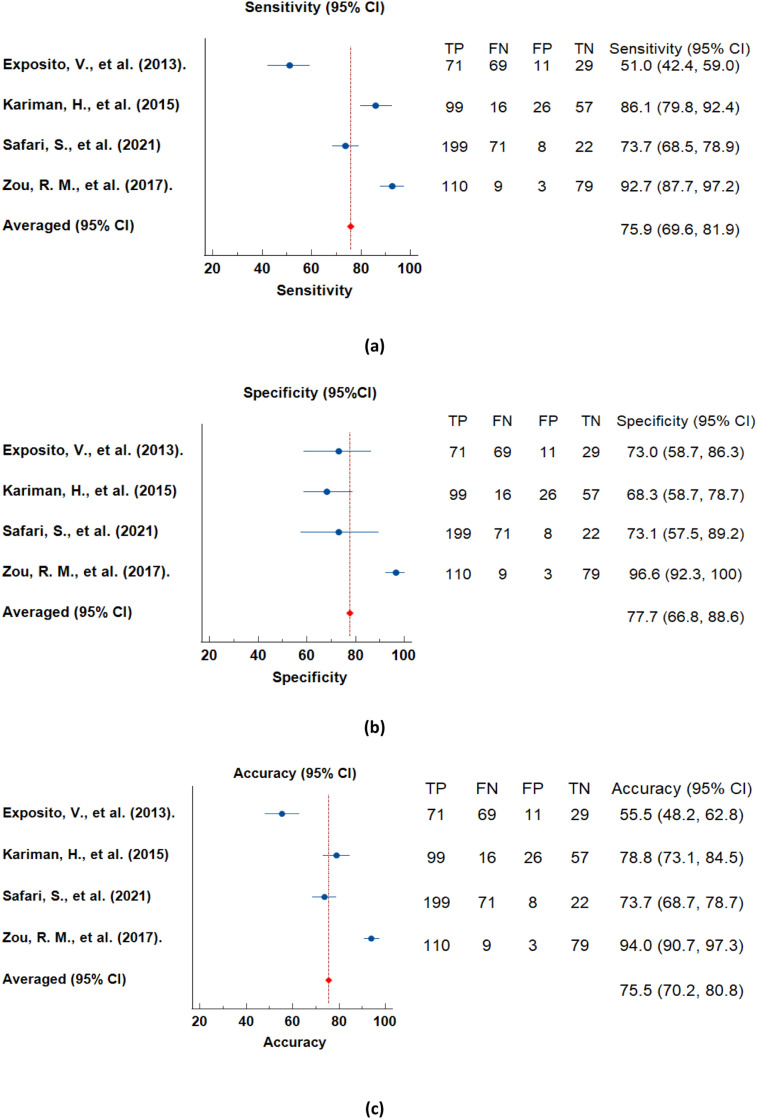
Forest Plot of Different Performance Metrics Estimate from the Studies Using Scoring Method in Syncope Classification. The forest plots, accompanied by 95% confidence intervals, visually depict the performance metrics—Sensitivity, Specificity, and Accuracy—of Machine Learning Algorithms employing scoring methods. This figure provides a comprehensive view of algorithmic efficacy in syncope classification.

Table 3 shows the characteristics of each study which used clinical risk score to predict syncope. The Calgary score, modified Calgary score, Calgary Syncope Symptom Score (CSSS), Evaluation of Guidelines in Syncope Study (EGSYS) and Canadian Syncope Risk (CSRS) were the clinical risk scores used to predict syncope occurrence. The range of sensitivity achieved by the clinical risk scores was 51–92.7% while the range for specificity was 57.32–96.6%. Accuracy was not mentioned by any of the four studies. The range of PPV was 57–87%. Zou et al. [32] achieved the highest sensitivity and specificity with a range of 91.46–92.7% and 95.8–96.6% by using the Calgary score and modified Calgary score. Exposito et al. [33] achieved the highest PPV by 87% using CSSS. The forest plot analysis of studies using scoring method is shown in Fig. 4.

**Table 3:** Characteristic of included scoring method studies.

Article	Subject		Age range	Parameters	Scoring Method	Performance Metrics			
	No. of Subjects (n)	No. of Males (n, %)				Sensitivity (%)	Specificity (%)	Accuracy (%)	PPV (%)
Exposito, V., et al. (2013) [33]	180	103(57.2)	>60	Bifascicular block/asystole /supraventricular tachycardia /diabetes, lightheaded spells or faint with prolonged sitting or standing, warm or sweating before a faint, lightheaded spells or fainting with pain or in medical settings, age>35, remember anything about being unconscious	Calgary syncope symptom score	51	73	55.5*	87
Kariman, H., et al. (2015) [37]	198	123(62.3)	13-98	Abnormal ECG, palpitations/dyspnea, syncope in supine position/effort syncope, age >64 years, precipitating and predisposing factors, prodromes, blurred vision, neurovegetative signs, precipitating and predisposing factors, autonomic prodromes	EGSYS	EGSYS-U: 86.08 EGSYS-M:91.30	EGSYS-U: 68.29, EGSYS-M: 57.32	78.8*	EGSYS-U: 77.78, EGSYS-M: 57
Safari, S., et al. (2021) [38]	300	194(64.7)	>18	Vasovagal symptoms, history of heart disease, blood pressure, troponin level, QRS duration, QRS axis, corrected QT interval, and the diagnosis made in emergency department	CSRS	73.68	73.05	73.7*	68.53
Zou, R. M., et al. (2017) [32]	201	95(47.26)	5-18	Waking with cut tongue, prodromal deja vu or jamais vu, loss of consciousness with emotional stress,head turning to one side during loss of consciousness, abnormal behaviour noted by bystanders, postictal confusion, presence of presyncope, diaphoresis before a spell, loss of consciousness with prolonged sitting or standing	Calgary score and modified Calgary score	91.46-92.7	95.8-96.6	94.0*	-

⁎ Accuracy is back-calculated, PPV: Positive predictive value; EGSYS: Evaluation of Guidelines in Syncope Study, EGSYS-U: Evaluation of Guidelines in Syncope Study-Univariate, EGSYS-M: Evaluation of Guidelines in Syncope Study-Multivariate, CSRS: Canadian Syncope Risk Score.

By comparing the ML algorithms and clinical risk scores in terms of performance metrics, ML algorithms were able to achieve higher sensitivities and specificities. The ML algorithms achieved the highest sensitivity and specificity values of 97.4% and 97.3%, respectively, while clinical risk scores achieved the highest sensitivity and specificity of 92.7% and 96.6% respectively. However, the highest performance metrics from ML algorithms were not obtained from the same classification algorithm while clinical risk scores were able to obtain the highest performance metrics within the same risk score, which was the Calgary and modified Calgary score.

The robust performance of ML algorithms, showcasing remarkable sensitivities and specificities, underscores their potential for enhancing syncope diagnosis. It's noteworthy that the versatility of ML algorithms, achieving peak metrics across diverse classification methods, introduces a dynamic aspect to their diagnostic capabilities, distinguishing them from the more consistent yet limited clinical risk scores within the Calgary and modified Calgary score frameworks.

## Discussion

Basic signals such as ECG and BP are important in syncope classification with all included studies collecting ECG signals and seven studies have recording BP during HUTT. PPG and carotid sinus massage are options for signal collection as only one of the studies collected each signal. ECG should be included within a general evaluation for syncope as the presence of an abnormal ECG indicates the possibility of cardiac syncope [34,35]. BP needs to be measured when HUTT is carried out at both the supine and upright position; continuous blood pressure monitoring is also significant during the assessment [1]. Orthostatic hypotension can be detected by continuously measuring the blood pressure during HUTT. HR, RRI, Left Ventricular Ejection Time (LVET), HRV, MBP, DBP and SBP are parameters that are extracted from the signals collected which play important roles in syncope classification.

The integration of signal acquisition and ML models constitutes a comprehensive and sophisticated approach to syncope classification, seamlessly blending conventional medical assessments with cutting-edge ML techniques. The researchers employ a diverse array of ML models (refer to Table 2), each contributing unique strengths to the classification of syncope. Notably, the SVM excels in discriminating n-dimensional vectors, making it well-suited for extensive patient datasets. K Nearest Neighbors places emphasis on the 'k' parameter for diagnostic efficacy, Multinomial Naïve Bayes leverages probabilistic learning, Gaussian Naïve Bayes assumes a Gaussian distribution, Logistic Regression excels in binary prediction, and Random Forest utilizes an ensemble of tree classifiers with randomized vectors. Meanwhile, Decision Tree employs a divide-and-conquer strategy. The collaborative utilization of these models offers a nuanced and sophisticated approach to the classification of syncope.

All the ML algorithms in ten articles were evaluated and all of them have a mean sensitivity of 88.4% (95% CI: 81.9–93.7%), mean specificity of 80.6% (95% CI: 73.7–87.7%) and mean accuracy of 85.8% (95% CI: 78.5–92.8%). The sensitivity, specificity, and accuracy after excluded the two studies has increased where, sensitivity become 89.1% (95% CI: 82.1–94.5%), specificity increased to 83.7% (95% CI: 77.5–89.3%) while accuracy become 86.4% (95% CI: 78.4–94.1%). All studies presented at least one model with performance of higher than 80% in classification of syncope. Klemenc et al. [22] reported that their statistical models using HRV spectral analysis and BRS data from the first 15 min of HUTT were not able to predict the test outcome and not useful for clinic prediction. The other nine machine learning algorithms were believed to aid clinical practice and can improve the diagnosis of syncope in the future [20]. Machine learning algorithms can help to reduce the examination time of HUTT and minimize the uncomfortable diagnosis duration to increase the efficiency of syncope units in the hospital or clinic. However, some studies stated their proposed algorithm were tested within a small population and further evaluation with a larger population and clinical records will be needed to verify the generalization of the proposed approach [18,31].

Models using ROC analysis achieved the highest sensitivity and specificity with 97.3 % for RR/SBP in separate articles. Miranda et al. [36] applied the ROC curve and considered the cardioinhibitory response as a stable variable, the area under the curve (AUC) of Low Frequency (LF) component obtained the optimal sensitivity and specificity. Mereu et al. [30] mentioned that all data for each subject and variable were subtracted from the tilting data, the average of the first three minutes, for isolating the variation over the tilting period and removing the influence of the initial value. By classifying true positive and false negative as HUTT+ group and false positive and true negative as HUTT- group, a ROC was constructed, and AUC of ROC were calculated.

Couceiro et al. [17] obtained 95.2 % sensitivity and 95.4 % specificity with their ML algorithm, both performance metrics higher than most of the studies. Couceiro et al. [17] mentioned that the main steps for the proposed algorithm involve detection of motion artifacts, parameter extraction and post processing, feature evaluation and syncope prediction. The PPG signal and ECG signal was collected for parameter extraction using extension of the algorithm and Pan-Tompkin's algorithm. A sliding window box plot analysis is used to remove the outlier from extracted parameters. There are two sets of features that are derived from five parameters, resulting in ten features. The relevance of each feature is assessed by the AUC of the ROC curve, while its redundancy was assessed by Spearman's Rank Correlation Coefficient. The Minkowski distance metric was used for assessment of the distance between the evolving trajectory and the stable orthostatic reference. The performance of the algorithm undergoes three phases of evaluation which is 3W-DS validation, 3W-DS test and leave-one-out validation to improve its performance.

Clinical risk prediction scores have been utilized as clinical decision aids to risk stratify those presenting to syncope in emergency settings [37]. Only a handful of scoring methods have been evaluated. The Calgary syncope symptom score is a simple point score that contains seven parameters based on historical features that identifies younger patients with vasovagal syncope with a high sensitivity and specificity [33]. According to Kariman et al. [37] EGSYS contain ten parameters that are designed for focusing on differentiating cardiac and non-cardiac syncope. Zou et al. mentioned that Calgary score and modified Calgary score have been used to distinguish epilepsy from neurally-mediated syncope in children, which are evaluated based on nine parameters collected. CSRS is a scoring system which has eight parameters to classify patients with syncope and predicts serious syncope-related outcomes during the following 30 days [38].

Machine learning algorithms perform better than clinical risk prediction scores for syncope. The best sensitivity-specificity compromise for ML algorithm was published by [17] and with the average prediction time of less than two minutes. However, the study also mentioned that the prediction time by using a three-way data split test was over four minutes. This duration is enough to guide the patient to carry out physical counterpressure maneuvers (PCMs) or simply sit down to counter the drop in BP, avoiding occurrence of syncope. The prediction model by Couceiro et al. used five parameters, which are HR, pulse arrival time, stiffness index, reflective index, and LVET. By comparing Couceiro et al. with all scoring methods, Couceiro et al. collected fewer parameters for syncope prediction. Thus, ML algorithms are able to shorten testing time for HUTT potentially removing the need for the patient to experience unpleasant symptoms.

The performance of ML algorithms can be improved from time to time. When ML algorithms keep gaining and learning new data, the accuracy and efficiency of prediction will improve. However, it requires larger and more complex datasets for training in order to achieve better prediction performance [39]. Mossello et al. [40] spent about three and half years collecting data from 372 subjects, which is time consuming and inefficient. The challenges of collecting large and complex data may to be solved by using free access medical research databases such as PhysioNet, which is able to reduce the time taken for collecting data. However, some of the data and parameter needed might not currently available in PhysioNet, which bring open access data sources become a future trend for collecting data.

None of the studies included measured impedance cardiography (ICG) that is able to provide important parameters such as cardiac output (CO). ICG is a non-invasive measure of changes in thoracic impedance generated by fluctuating blood volume during cardiac cycle, allows calculation of stroke volume and cardiac output [41]. The fall of BP during vasovagal syncope is mediated initially by decreased CO and reduction of CO may be the primary cause of the hypotension of vasovagal syncope, hence the use of ICG may improve the predictive value of ML algorithms, which might be tone of the important parameter in future study of syncope prediction.

One of the limitations of this systematic review is the subject's age in most studies didn't have a clear cut or specific age range. The age range of Zhang et al. is 5-17, consider as paediatric patients while Mossello et al. chose patient older than 65 as subject. Obviously, performance metric of both studies should not be compared as the characteristic of age of both studies are not same. Other studies also didn't have a clear cut on the age range as the subject is mixed with adolescent, teenager, adult and elder. Therefore, when the algorithm applied on patient, the performance might be unsatisfied.

Most of the study except [17,18,20,22] did not mention their validation test for the constructed algorithm. As in the current ML papers, 80–20 or 70–30 train-validation split is a standard practice to assess the bias of model and address overfitting. Thus, lack of the detail of validation process is one of the limitations of this study. Studies should mention their process of constructing algorithm to let reader to have a better understanding on the concept of algorithm, preventing insufficient information for referencing.

This systematic review is limited by the absence of performance metrics from many studies. In addition, the heterogeneity in HUTT protocols within the studies included also made comparison between studies a major challenge. Different studies used different duration (ranging from 5–30 minutes in supine rest and 20–45 minutes in tilting) and position for HUTT (60–80 degree of tilting). Further studies should consider incorporating larger dataset or pooling existing datasets. Nevertheless, the use of ML algorithms does show promise in reducing the time consumption and unpleasant symptoms associated with HUTT, which will potentially also serve to increase testing capacity and enhance accessibility to accurate syncope diagnostic processes. Another limitation of this review is the selected study did not carry out any comparison of their ML algorithm with the existing standard of care, where they only focus on the performance metric of the ML algorithm. Comparison between classification algorithm and the existing standard of care is important for evaluating their prediction model whether the diagnosis time and performance able to meet the basic requirement.

## Conclusion

ML algorithms are able to achieve an average sensitivity of 88.8% and average of specificity of 81.5% while clinical risk scores achieved 75.9% sensitivity and 77.7% specificity. Besides that, ML studies used minimal parameters for classification, which is as low as five parameters. In conclusion, the ML algorithm can predict syncope accurately with fewer parameters needed and better performance metrics. Our result could be used as a reference for techniques and parameters for the development of syncope prediction models. In addition, ML algorithms could be improved from time to time with larger data sets, with the use of ML algorithms demonstrating the potential of reducing testing time and removing the requirement to invoke potentially unpleasant symptoms with downstream effects of increasing testing capacity and accessibility.

## CRediT authorship contribution statement

**Choon-Hian Goh:** Conceptualization, Methodology, Data curation, Software, Writing – original draft, Writing – review & editing, Validation, Supervision. **Mahbuba Ferdowsi:** Methodology, Software, Writing – original draft, Writing – review & editing, Validation. **Ming Hong Gan:** Methodology, Data curation, Software, Writing – original draft, Writing – review & editing, Validation. **Ban-Hoe Kwan:** Conceptualization, Writing – review & editing, Validation, Supervision. **Wei Yin Lim:** Conceptualization, Methodology, Data curation, Writing – review & editing, Validation. **Yee Kai Tee:** Writing – review & editing, Validation. **Roshaslina Rosli:** Writing – review & editing, Validation. **Maw Pin Tan:** Conceptualization, Methodology, Software, Writing – original draft, Writing – review & editing, Validation.

## Declaration of Competing Interest

The authors declare that they have no known competing financial interests or personal relationships that could have appeared to influence the work reported in this paper.

## Data Availability

No data was used for the research described in the article.
